# Alkyl-Quinolones derivatives as potential biomarkers for *Pseudomonas aeruginosa* infection chronicity in Cystic Fibrosis

**DOI:** 10.1038/s41598-021-99467-7

**Published:** 2021-10-20

**Authors:** Serge Michalet, Pierre-Marie Allard, Carine Commun, Van Thanh Nguyen Ngoc, Kodjo Nouwade, Bruna Gioia, Marie-Geneviève Dijoux-Franca, Jean-Luc Wolfender, Anne Doléans-Jordheim

**Affiliations:** 1 Université de Lyon, Lyon, France; 2 Université Claude Bernard Lyon 1, Lyon, France; 3 Research Group on Environmental Multiresistance and Bacterial Efflux, UMR CNRS 5557 Ecologie Microbienne, Université Claude Bernard Lyon 1, CNRS, VetAgro Sup, ISPB, Villeurbanne, France; 4 School of Pharmaceutical Sciences and Institute of Pharmaceutical Sciences of Western Switzerland, University of Geneva, CMU, Rue Michel-Servet 1, 1211 Geneve 4, Switzerland; 5 Research Group on Bacterial Opportunistic Pathogens and Environment, UMR CNRS 5557 Ecologie Microbienne, Université Claude Bernard Lyon 1, CNRS, VetAgro Sup, ISPB, Villeurbanne, France; 6 EA 4446, Molécules bioactives et chimie médicinale (B2MC), ISPB-Faculté de Pharmacie, Lyon, France; 7 Laboratoire de Bactériologie, Institut des Agents Infectieux, Hospices Civils de Lyon, Lyon, France

**Keywords:** Clinical microbiology, Microbiology, Biomarkers, Diagnostic markers, Metabolomics

## Abstract

In Cystic Fibrosis (CF), a rapid and standardized definition of chronic infection would allow a better management of *Pseudomonas aeruginosa* (*Pa*) infections, as well as a quick grouping of patients during clinical trials allowing better comparisons between studies. With this purpose, we compared the metabolic profiles of 44 in vitro cultures of *Pa* strains isolated from CF patients at different stages of infection in order to identify metabolites differentially synthetized according to these clinical stages. Compounds produced and secreted by each strain in the supernatant of a liquid culture were analysed by metabolomic approaches (UHPLC-DAD-ESI/QTOF, UV and UPLC-Orbitrap, MS). Multivariate analyses showed that first colonization strains could be differentiated from chronic colonization ones, by producing notably more Alkyl-Quinolones (AQs) derivatives. Especially, five AQs were discriminant: HQC5, HQNOC7, HQNOC7:1, db-PQS C9 and HQNOC9:1. However, the production of HHQ was equivalent between strain types. The HHQ/HQNOC9:1 ratio was then found to be significantly different between chronic and primo-colonising strains by using both UV (*p* = 0.003) and HRMS data (*p* = 1.5 × 10^–5^). Our study suggests that some AQ derivatives can be used as biomarkers for an improved management of CF patients as well as a better definition of the clinical stages of *Pa* infection.

## Introduction

In Cystic Fibrosis (CF), the dysfunction of the CFTR channel is responsible for the generation of a viscous mucus in many organs^1^. This mucus obstructs the airways leading to repetitive pneumonia and lung function decline. Many bacteria have been identified as pathogens of CF lungs. Pulmonary infections due to one of the most feared micro-organism in this context, *Pseudomonas aeruginosa* (*Pa*), are among the primary causes of morbidity and mortality in CF patients^2,3^. As an example, airway infections with *Pa* are associated with higher rates of FEV1 decline in CF patients^4^.

The progression of *Pa* infection is characterized in three stages from initial to intermittent and chronic^5,6^, according to the different CF clinical situations. This evolution is associated with phenotypic and genotypic changes in *Pa* strains, evolving into a pattern of persistence or chronicity. These changes result in the appearance of highly resistant and partially or completely depigmented strains. In addition, the formation of biofilms is a very common phenomenon in these infections as well as the development of a mucoid phenotype^7^. From a genetic point of view, it has been shown that the genome of chronic strains is highly mutated, allowing the adaptation to the pulmonary environment^8^. This makes, in turn, the eradication of these strains difficult or impossible. Thus, it is now clearly demonstrated that chronic infections by *Pa* represent an important risk of adverse evolution for the patient influencing its survival, with decline of lung function and increased exacerbations, antibiotic use and hospitalizations^9^.

During clinical practices, it would be interesting to be able to rapidly define and characterize the stage of colonisation / infection in order to better adapt the respiratory sampling and analysis, the therapeutic management of the patients and to develop strategies to counteract this implantation. In addition to this benefit, chronic infections represent commonly used indicators of CF disease progression^10,11^. Moreover, a standardized definition of chronic *Pa* infection is of great importance within clinical care as it would allow a rapid grouping of patients during clinical trials, as well as relevant comparisons between studies and laboratories^10,11^.

Today, infections are generally classified with Leed’s criteria, i.e. based on number of sputa colonised by *Pa* in one year. As an example, chronic colonisations are defined as 50% of positive *Pa* cultures during the preceding 12 months^6^. Other definitions exist, taking into account new criteria such as serology^11^. However, according to all these definitions, at least 6 months are needed before a chronic infection can be determined, limiting therapeutic follow-up of CF patients. Rapid identification of these infections is therefore a priority in the management of this disease. In this way, recent approaches including quantitative PCR are now developed^9^, but they still need evaluation and standardisation.

One of the possible strategies to monitor *Pseudomonas* implantation and progression is based on metabolomics. Indeed, *Pa* excretes a large amount of secondary metabolites including virulence factors, siderophores and signalling molecules that allow this pathogen to efficiently colonize the respiratory tract and/or to inhibit the growth of other bacteria^12–15^. The metabolome of *Pa* is diverse and variable in lungs, changing over time and depending on interactions with environment and microbiota conditions^16–20^. This could be partially explained by the diversity of Quorum Sensing (QS) regulation systems expressed in *Pa*^7,21^. Among the signal molecules regulating QS, *Pa* produces more than 50 derivatives of 2-alkyl-4-(*1H*)-quinolones (AQs) through the Pqs-ABCDE biosynthetic pathway^22,23^. Their diversity, which is generated through PqsBC, arises from the length of the alkyl chain, the presence of unsaturation or nitrogen oxidation^24^. AQ derivatives, such as 2-heptyl-3-hydroxy-4(1*H*)-quinolone (referred to as the *Pseudomonas* quinolone signal PQS), its precursor 2-heptyl-4(1*H*)-quinolone (HHQ), and their C9-congeners 2-nonyl-3-hydroxy-4(1*H*)-quinolone (C9-PQS) and 2-nonyl-4(1*H*)-quinolone (NHQ), have been shown to be signal molecules involved in cell-to-cell communication and in the regulation of *Pa* virulence factors^25–28^. They have also been described as antimicrobial agents, especially against *Staphylococcus aureus*^29–32^, and they also display diverse biological functions such as antioxidants and iron chelators^33,34^. AQs are categorized into 5 different subclasses^35^: 2-alkyl-4-(1*H*)-quinolones (HQ derivatives; Series 1), 2-alkenyl-4-(1*H*)-quinolones (dbHQ derivatives, Series 2) and their N-oxyde congeners (HQNO derivatives and dbHQNO derivatives respectively, Series 4 and 5 respectively) and 2-alkyl-3-hydroxy-4(1*H*)-quinolones (PQS derivatives, Series 3). Previous studies pointed out that these molecules are detected in *Pa*-infected CF patients^16,35–38^ and that some of these AQs could represent potent biomarkers of *Pa* infection in CF patients^35,37^.

In this context, the objective of our study was to compare the metabolic profiles, and more particularly the AQs production, of 44 Pa strains isolated from 34 CF patients at different stages of infection and cultivated in vitro, in order to identify discriminant metabolites that would be differentially produced according to *Pseudomonas* phenotype (first-colonisation, intermittent colonisation or chronic infection). The evaluation of the relevance of the identified molecules as potential biomarkers of *Pa* phenotypes in CF patients is also discussed.

## Results

### UV-based metabolic profiling can discriminate between chronic and first colonisation *P. aeruginosa* strains

We collected 44 strains from the hospital bacteriology laboratory of the Institute of Infectious Agents (Lyon, France) (Table 1).

**Table 1:** Distribution of *Pa* strains according to the colonisation status of CF patients.

Colonisation status of the CF patients	Number of patients	Number of strains	Strains abbreviations
Primo-colonisation (never colonised before)	9	9	PP
Free (not colonised during at least one year before)	10	10	PF
Intermittently colonised	5	5	PI
Chronically colonised	10	20 (10 couples)	PC (non mucoid strains) PCM (mucoid strains)
Total	34	44	

They were isolated from the sputum of 34 CF patients, followed in the two CF centres of Lyon, and classified according to the clinical status of the patients (Leed’s criteria)^6^. Nine strains were recovered from 9 CF patients who were never colonised by *Pa* before this primo-colonisation (strains PP), 10 strains from 10 CF patients who were free from *Pa* at least one year before the isolation of the strain (strains PF), 5 strains were recovered from 5 patients with intermittent infection, defined as positive microbial culture in at least one and less than 50% of the sputa collected in the last year (strains PI) and 20 strains recovered from 10 patients with chronic infection (presence of *Pa* in at least 50% of the sputa analysed in the last twelve months). These 20 strains were divided into 10 pairs. Each pair, isolated from the same sample, contained a non-mucoid *Pa* strain (strains PC) and a mucoid *Pa* strain (strains PCM). To note, two chronically colonised siblings were included in this study with PC and PCM strains. Each strain was previously typed by Pulsed Field Gel Electrophoresis^39^ and showed different profiles except the strains corresponding to each couple and siblings (Fig. S1).

The analysis by UV method (UHPLC-DAD ESI/QTOF) of the organic extracts of the 44 supernatants obtained from the liquid culture of these 44 strains allowed to integrate a total of 97 UV peaks that were aligned into a matrix.

**Figure 1:**
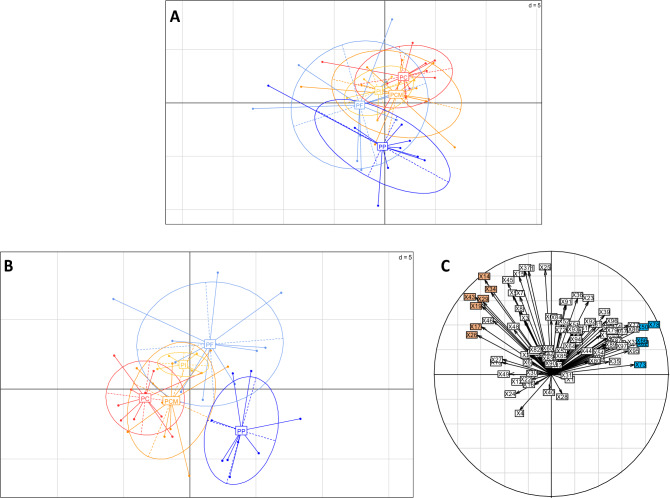
(**A**) PCA (axis 1: 19.6%; axis 2: 13.1%) achieved on 97 integrated UV peaks at 280 nm. The extracts obtained from the 44 strains isolated from 34 CF patients are displayed according to strain types: PP (strains from patients never colonised by *Pa*; dark blue), PF (strains from patients not colonised by *Pa* during at least one year before; blue); PI (patient intermittently colonised by *Pa*; yellow), PC and PCM (patient chronically colonised by *Pa*; red and orange respectively). (**B**) PLS-DA (axis 1; axis 2) obtained from 97 integrated UV peaks at 280 nm according to strain types. The extracts obtained from the 44 strains PP, PF, PI, PC and PCM. (**C**) Correlation circle (axis 1; axis 2). Colored peaks correspond to discriminant peaks between first colonisation strains (PP/PF) and chronic strains (PC/PCM), in blue for those that are more detected in first colonisation strains and in orange for those that are more detected in chronic ones.

A Principal Component Analysis (PCA) showed a distinction between strain types (Fig. 1A).

According to axis 2, the 9 first-colonisation *Pa* strains (PP) were grouped into a clearly separate area from the strains recovered from expectorations of patients with Pa chronic colonisation (PC and to a lesser extent the mucoid strains PCM). The other strain types (PI and PF) are ranging in between, with a great variability for PF strains. In order to identify discriminating peaks responsible for the segregation of strain types, a Partial Least Square-Discriminant Analysis (PLS-DA) was performed and allowed a better separation between strain types with PP strains being separated from PC and PCM according to axis 1 (Fig. 1B). Intermediate *Pa* (PI) were separated from PP and PC strains but overlapped with some PCM and PF strains. Chronic mucoid strains (PCM) were close to non-mucoid ones (PC) but showed a higher variability. This was also the case of PF strains which were not distinguishable from the other strain types, although they explained most of the variance of axis 2.

### Early stage colonisation strains produce more of some AQ derivatives

According to the analysis of the correlation circle (Fig. 1C), 12 peaks were selected and further confirmed to be discriminant between *Pa* strain types by monovariate analyses (Table 2).

**Table 2:**
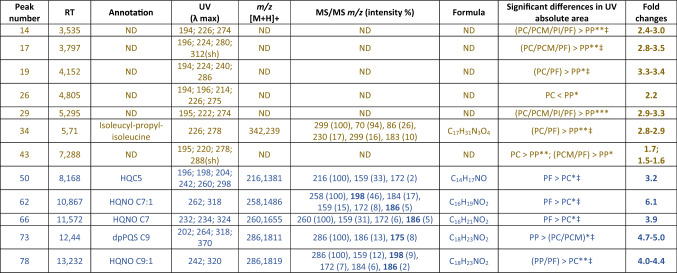
Discriminant peaks detected in extracts and also present (brown) or absent (blue) in culture medium, with their putative identity.

Significant differences in peak areas between strains are highlighted and *p* values obtained after ANOVA and post-hoc Tuckey’s HSD (or Kruskal–Wallis for non-parametric data^‡^) are shown for UV data (***p < 0.0001; **p < 0.001; *p < 0.05).

Compounds were identified according to their UV, HRMS and HRMS/MS spectra and compared with literature^40,41^. Among these, 7 compounds were detected in the growth medium (peaks in brown in Table 2) and 5 were identified as AQ derivatives (peaks in blue in Table 2) belonging to 3 different subclasses (PQS, HQ and HQNO). Looking at the significant differences between strain types, the 7 compounds present in the growth medium (including one compound annotated as a peptide derivative) were all found at higher levels, especially in chronic strains (PC), compared to primo-colonising (PP) ones for which these compounds were detected at a lower level in each case (Table 2). Concerning 5 AQ derivatives, 3 short chain AQs (i.e. HQC5, HQNOC7 and HQNOC7:1) were detected at higher levels only in PF strains compared to PC strains, and 1 compound (dbPQSC9) was found at a significantly higher levels only in PP strains as compared to PC/PCM. The last molecule identified as HQNOC9:1 was found at higher levels in PF and PP strains compared to PC strains. This compound was the one for which the difference was the most significant between PP/PF and PC strain types.

**Figure 2:**
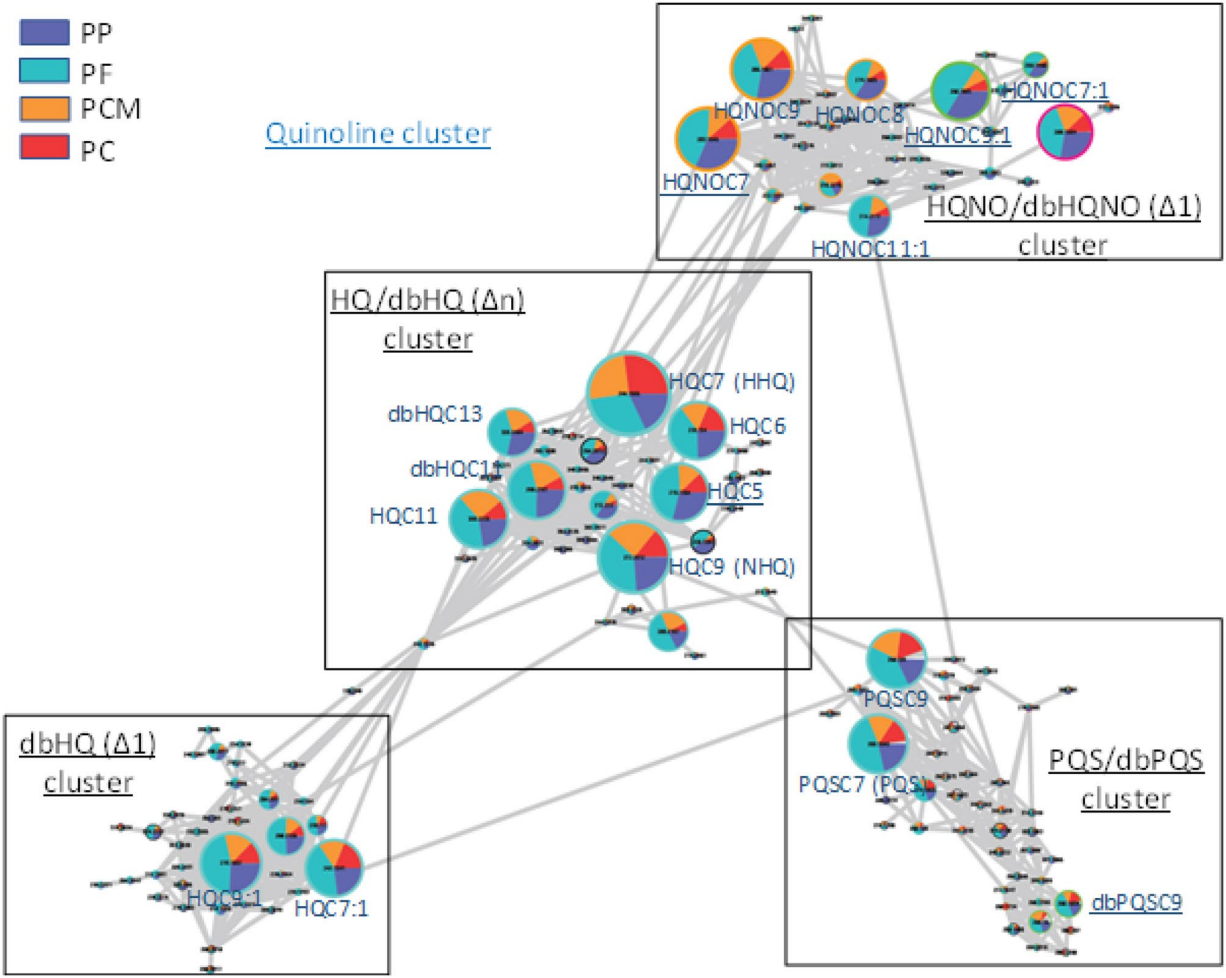
Quinoline cluster divided into 4 subclusters corresponding to different AQs classes : PP (strains from patients never colonised by *Pa*; dark blue), PF (strains from patients not colonised by *Pa* during at least one year before; blue); PC and PCM (patient chronically colonised by *Pa*; red and orange respectively).

The analysis of the second subset of extracts by UPLC-Orbitrap and molecular networking confirmed the higher production of AQs by early stage colonisation strains except for HHQ and allowed a more global view of the variations occurring between strains’ metabolisms. With this method, the main ions detected were divided into two big clusters which were annotated as peptide and quinoline derivatives respectively (Fig. S2). In the peptide cluster, the main ions detected were found at low levels in PP strains compared to the other ones, whereas in the quinoline cluster most of the ions detected were detected at higher levels in PF strains (and to a lesser extent also in PP ones) compared to chronic isolates (PC and PCM). In detail, this cluster can be divided into 4 sub-clusters (Fig. 2, Figs. S3–S6), for which PF strains and to a lesser extent PP strains produce more of these compounds compared to chronic ones.

This is particularly true for HQNOC9:1, the most discriminant metabolite according to UV analysis. However, a few of these AQ derivatives were found also in high proportions in chronic strains. This is the case of HHQ (HQC7) which was the AQ derivative found in the highest proportions in PC and PCM strains, close to or higher than the levels detected in PF or PP strains respectively (Fig. S3).

### HHQ/HQNOC9:1 ratio as a metabolic bio-indicator of chronic colonisation

**Figure 3:**
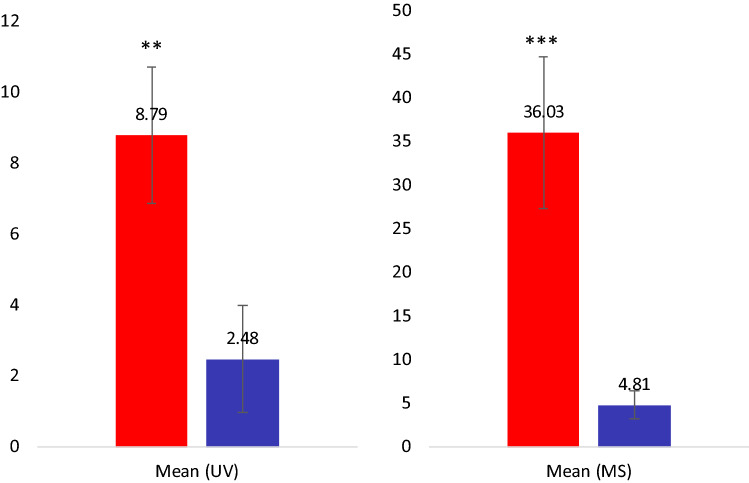
Mean HHQ/HQNOC9:1 ratio ± SEM detected in chronic strains (PC + PCM, n = 20; in red) and in first colonisation strains (PP + PF, n = 19; in blue), using both UV (left) and HRMS (right) data. Significant differences were found by using Kruskal–Wallis tests (chi-squared = 8.7066, df = 1, p-value = 0.003171 for UV, and chi-squared = 18.723, df = 1, p-value = 1.511 × 10^–5^ for HRMS).

Observing that in chronic strains, HHQ concentration is very important compared to HQNOC9:1, whereas in primo-colonising ones the concentration of HQNOC9:1 tends to be closer to that of HHQ, we hypothesized that the ratio HHQ / HQNOC9:1 could be a potential indicator of *Pa* phenotype in CF infection stage. Indeed, a significant difference in the HHQ/HQNOC9:1 ratio between chronic (PC + PCM, n = 20) and primo-colonising (PF + PP, n = 19) strains was observed by using both UV (*p* value = 0.003) and HRMS data (*p* value = 1.5 × 10^–5^) (Fig. 3).

To note, HRMS being more sensitive than UV, ratio was higher with this method, although AQ types may present different ionisation patterns depending on their concentration and on matrix effects^42^. However, it was not possible to determine an absolute threshold value, enabling to discriminate with certitude all chronic strains from primo-colonising ones since a few strains had deviant values.

## Discussion

Given the clinical and therapeutic impact of a chronic implantation of *Pa* in the lungs of CF patients, a diagnostic method capable of differentiating quickly the different phenotypes of *Pa* and the different stages of the infection, would be a major asset in the management of this disease, including the antibiotic strategy. Moreover, such a tool could lead to a universally accepted definition of chronic *Pa* infection, essential for clinical trial outcome measures^43^.

During lung colonisation, mutations occur in the genome of *P. aeruginosa* in order to allow the adaptation of this bacterium to the pulmonary environment. However, its gene expression is strongly associated to the activation of many transcriptional regulatory systems^44^. Consequently, correlations between genotype and phenotype are difficult to establish and the simple search for a mutation is not sufficient to characterize the metabolic changes that occur during long-term infection, and then to define the colonisation status of the patient. Metabolic approaches, considering these post-transcriptional regulations, appear relevant in the differentiation of *P. aeruginosa* phenotypes^44^.

In our study based on an in vitro culture of different strains of *Pa* isolated from CF patients, the unsupervised comparison of UV and MS-based chromatographic profiles of the organic extract obtained from strains’ supernatants, allowed to identify secondary metabolites that were differentially produced according to the clinical stage of infection. The method of extraction of the supernatant achieved in this study includes the use of two solvents: ethyl acetate (AcOEt) and dichloromethane (DCM), whereas in literature AcOEt is often used solely^16^. Preliminary experiments achieved with 4 strains (2 PP and 2 PC) aiming at comparing AcOEt and DCM extracts showed that although middle chain AQ derivatives (C_7_–C_10_) were similarly extracted by both solvents, long chain AQ (> C_10_) and short chain AQ (< C_7_) were better extracted with DCM or with AcOEt respectively (data not shown). The same tendency was observed for other metabolites like pyocyanin and phenazine derivatives that were better extracted with DCM or AcOEt respectively. The extraction method retained for the analysis of the 44 strains then included both solvents in order to get a better overview of metabolites produced by different strain types.

The results showed that AQs represent the main secondary metabolites detected in the organic extract of strains’ supernatant, both in UV- and MS-based analyses, and among these, some were discriminant between strains. These molecules are produced through PQS systems from the condensation of anthranilate with fatty-acids, and not with β-keto fatty acids as it was initially proposed^45^. A variability of these metabolites can be observed, distinguishing more particularly primo-colonising strains (PP and PF) from chronic ones (PC and PCM), the former producing more of most AQs detected. The overlap of PI strains with PF and PCM strains in Fig. 1B suggests similar behaviours potentially due to a common adaptation step in lungs or in their respective reservoirs in upper airways with PI behaving as a metabolic intermediate between PF and PCM. However, the PFGE-signature reveals major genetic differences between the strains, showing that they are genetically clearly different.

As expected, strains related to first colonisation (PP strains) appear more metabolically active (i.e. using more efficiently the carbon resources provided by the culture medium) than other types of strains. However, an interesting point is the fact that strains from patients “never” (PP strains) and patients “free” (PF strains) showed some similarities, especially concerning the diversity and relative quantity of AQs produced, as they both showed a significant overproduction of these AQs compared to chronic strains. These observations are in line with results from previous studies pointing at the differences between *Pa* strains isolated from first and chronic lung colonisations, due to the adaptive fitness of these strains during disease evolution^7,46^. It is noteworthy that PF strains were not able to use carbon sources from the culture medium in the same was as PP strains did. This could suggest that adaptation and modifications of the metabolic capacities as carbon utilization occurs very early in the disease progressions. Further, UV-based profiling allowed to show a great variability among PF strains profiles that was not retrieved with PP strains. These differences in the metabolism of both strains shows that although they both synthetize a high quantity and diversity of AQs compared to chronic strains, they possess distinct metabolism and may thus present different evolutions after lung infection of CF patients. This overproduction of some AQs could be linked to the "invasive" phenotype of PP and PF strains, since these bacteria have to implant in CF lungs and to colonize an environment already invaded by other microorganisms, unlike PC strains which harbour a modified phenotype using less carbon sources (or slower) and synthetizing at lower levels most AQ derivatives, thus allowing their persistence. These chronic strains down-regulate the expression of some factors (bacterial replication, pigments expression, flagellar or pilus motility, secreted protease activity…) and/or lose them to the benefit of others (biofilm formation) in order to escape the immune defences and to resist harmful pressures from the host environment and antibiotics^7,47,48^. AQs, being multifunctional molecules, involved in cell-to-cell signalling, resistance to oxidative stress or metal chelation but also in polymicrobial interactions (bacterial and fungal) or with the host, it seems obvious that their production varies during pathogenesis^18,33^. The results concerning the compound HQNOC9:1 could illustrate this hypothesis. In our study, we found that HQNOC9:1 was produced in significantly larger quantities by both PP and PF strains compared to chronic ones. Besides, HQNOC9:1 has been shown to inhibit the growth of other bacteria, especially *S. aureus*^49^. The production of this compound could then be related to a more virulent phenotype of *Pa* at the beginning of the pulmonary infection and/or the need for *Pa* to interact with other bacteria in order to settle permanently in the lungs. On the other hand, all AQs are not only produced at the lungs’ invasion stage as HHQ was found to be more produced by chronic strains. This result is consistent with those of Barr et al.^35^*.* Studying correlations between AQs detection in biological fluids and the clinical status of infection of CF patients, these authors observed that HHQ detected in urine, plasma and sputum showed the best correlation levels with the clinical status of CF patients (adults and children) considered as currently infected by *Pa* or not among 6 AQs tested^35^. Further, the authors detected a high concentration of this compound in plasma and urine of patients considered as chronically infected (from 1 to 3 log more than its level of detection in patients free or never). From this and from the results of our study, we could then hypothesize that chronic strains tend to keep a high level of HHQ production in situ. This high production could be explained by the fact that HHQ is strongly related with biofilm formation in *Pa*^50^, a phenotype well described during chronic stage.

The mucoid phenotype is a factor often described as related to chronic infections, even if this character is not always present during the late phase^5^. According to our study, whether this factor is present or not does not interfere with the grouping of strains’ metabolic profiles according to the colonisation stage.

Our study has limitations due in part to the low number of strains in each group. An analysis of a larger number of isolates is now required to increase the strength of our results. Moreover, different media and growth conditions (especially biofilms growth) should be evaluated since the *Pa* production of different compounds is dependent on available precursors. In addition, our analysis is based on strains, so it remains dependent on the culture stage. It is well known that cultures are sometimes not sensitive enough (limiting detection in *Pa*-poor samples) especially when antibiotics are present. For all these reasons, a culture-independent method is needed. Most AQs detected in our study have also been detected in lungs, sputa, blood, and urine of *Pa*-infected CF patients, indicating that these molecules are produced by *Pa *in vivo^16,35–37^*.* We could then imagine to use the ratio HHQ / HQNOC9:1 by dosing these products directly in biological fluids, especially those obtained by non-invasive techniques. This would allow a more clinically relevant and rapid assay, independent from culture conditions but limited to expectorating patients (for patients with swab only, culture step will still be needed). Webb et al. recently demonstrated that none of the different AQs measured in patients’ fluids (including HHQ and HQNO) were significantly associated with a decline in clinical symptoms relative to disease evolution in CF patients^38^. Our next objective is to evaluate this, using the HHQ/HQNOC9:1 ratio rather than one independent marker. Indeed, this indicator could increase the sensitivity of the methodology compared to the use of one metabolite alone and would then enable a rapid classification of *Pa* strains.

Using the analysis of the AQs produced by *Pa* during CF pulmonary infection as biomarkers could lead to an improved management of CF patients as well as a better definition of the different clinical stages of infection. The development of biosensors could facilitate the implantation of such technic among the diagnostic tools, and seems to constitute an important step for routinely implementation of this approach, both for economic and technical reasons^51^. The implementation of a metabolic approach could also lead to a better understanding of the physiopathology of the disease and then let us imagine new approaches to avoid CF lungs’ colonisation by *Pa* and / or to eradicate this bacterium from the lower airways.

## Material and method

### Pa strains and growth conditions

Forty-four strains, isolated from the sputum of 34 CF patients were recovered from the hospital bacteriology laboratory of the Institute of Infectious Agents (Lyon, France) (Table 1). Each isolate was classified according to the clinical status of the patients (Leed’s criteria) and then, stored at − 20 °C before use.

*Pa* strains were cultured on agar LB and incubated overnight at 37 °C. Colonies were suspended on LB liquid medium and OD_600nm_ was adjusted to 1. Each suspension was diluted 1/100 and incubated at 37 °C for 24 h under agitation (125 rpm). The supernatant was recovered after centrifugation at 4000 rpm and filtering at 0.45 µm and 0.22 µm. About 20 mL of supernatant was prepared for each strain.

Each strain was previously typed by Pulsed Field Gel Electrophoresis^39^.

### Extraction of supernatant

Bacterial supernatant was extracted with dichloromethane (DCM) and with ethyl acetate (AcOEt) (two times each) in 30 mL conic glass tubes (Verrerie Villeurbannaise, Villeurbanne, France). For that, 13 mL and 8 mL of DCM were successively added to 13 mL of bacterial culture supernatant, vortexed 1 min and centrifuged 10 min at 3500 rpm. After recovery of both DCM phases, the same procedure was repeated with AcOEt. Organic phases obtained following the DCM and the AcOEt extractions were pooled, and two aliquots were prepared and concentrated to dryness under reduced pressure (CentriVap, Labconco, USA) before being stored at − 20 °C until analysis. Samples were concentrated in methanol (MeOH) at 10 mg/mL before analysis. One separate aliquot of each sample’s extract was used for both type of chromatographic analysis.

### Chromatographic analysis

#### UHPLC-DAD-ESI/QTOF, UV metabolite profiling and compound identification

A subset of the 44 extracts obtained was analysed by UHPLC-DAD ESI/QTOF (Agilent 1290 infinity equipped with a Diode-Array Detector and linked with an Agilent Quadrupole Time Of Flight 6530, using a Poroshell 120 EC-18 column (2.7 µm, 3.0 × 100 mm, Agilent) and a pre-column (Poroshell 120 EC-18 (2.7, 3.0 × 15 mm, Agilent) with a gradient of 0.1% formic acid in water (A) and acetonitrile (B) as follows: 1% of B from 0 to 1 min and increasing with a linear gradient to 55% of B at 15 min and 100% of B at 16 min for 1 min^52^. All solvents were LC–MS grade (Optima). The flow rate was adjusted at 1.2 mL/min and the injection volume was 2 µL. UV spectra were recorded between 190 and 600 nm. The ESI source and QTOF spectrometer were optimized as follows: positive ionization in auto-MSMS mode, scan spectra from *m/z* 100 to 2000 at 2 GHz, capillary voltage 3.5 kV, fragmentor 120 V, fixed collision-induced dissociation (CID) energy at 20 eV. Nitrogen was used as the nebulizing gas with a flow rate of 12 L/min at 310 °C and 40 psi^52^. The mass detector was calibrated before analysis by mass check with calibrating standards according to the constructor’s instructions.

UV chromatograms recorded at 280 nm were integrated using MassHunter Qualitative Analysis B.07.00 (Agilent). The chromatogram of the Quality Control (QC) sample (corresponding to a homogenous mix of all 44 extracts) was used to homogeneously integrate 97 peaks that were aligned into a matrix and expressed as absolute area. Data were treated by multivariate (PCA, PLSDA) and monovariate (ANOVA and Tuckey’HSD or Kruskal Wallis) analyses using RStudio software v 3.3.0 (packages ade4, mixomics, RVaidememoire).

Compounds were annotated by analysis of their UV, HRMS and HRMS/MS spectra using MassHunter Qualitative Analysis (Agilent). Matching formula were searched on Scifinder to confirm the identity and MSMS spectra were compared to literature for AQ derivatives^40,41^.

For HHQ/HQNOC9:1 ratio, both integrated UV peaks and integrated EIC (at *m/z* = 244.169 and *m/z* = 286.1819 for HHQ and HQNOC9:1 respectively) were used to calculate the mean and standard error to the mean (SEM) for chronic strains (i.e. PC and PCM strain types) and primo-colonising ones (i.e. PF and PP strains).

#### UPLC-Orbitrap, MS data pre-treatment and molecular network construction

The second subset of the 44 extracts obtained were analysed on a Waters Acquity UPLC system interfaced to a Q-Exactive Focus mass spectrometer (Thermo Scientific, Bremen, Germany), using a heated electrospray ionization (HESI-II) source. Thermo Scientific Xcalibur 3.1 software was used for instrument control. The LC conditions were as follows: column, Waters BEH C18 50 × 2.1 mm, 1.7 μm; mobile phase, (A) water with 0.1% formic acid; (B) acetonitrile with 0.1% formic acid; flow rate, 600 μL min^−1^; injection volume, 6 μL; gradient, linear gradient of 5–100% B over 7 min and isocratic at 100% B for 1 min^53^. The optimized HESI-II parameters were as follows: source voltage, 3.5 kV (pos); sheath gas flow rate (N_2_), 55 units; auxiliary gas flow rate, 15 units; spare gas flow rate, 3.0; capillary temperature, 350.00 °C, S-Lens RF Level, 45. The mass analyzer was calibrated using a mixture of caffeine, methionine−arginine−phenylalanine−alanine−acetate (MRFA), sodium dodecyl sulfate, sodium taurocholate, and Ultramark 1621 in an acetonitrile/methanol/water solution containing 1% formic acid by direct injection. The data-dependent MS/MS events were performed on the three most intense ions detected in full scan MS (Top3 experiment). The MS/MS isolation window width was 1 Da, and the stepped normalized collision energy (NCE) was set to 15, 30 and 45 units. In data-dependent MS/MS experiments, full scans were acquired at a resolution of 35 000 FWHM (at *m/z* 200) and MS/MS scans at 17 500 FWHM both with an automatically determined maximum injection time. After being acquired in a MS/MS scan, parent ions were placed in a dynamic exclusion list for 2.0 s^53^.

The MS data were converted from .RAW (Thermo) standard data format to .mzXML format using the MSConvert software, part of the ProteoWizard package^54^. The converted files were analysed using the MzMine software suite v. 2.38^55^.The parameters were adjusted as follows: the centroid mass detector was used for mass detection with the noise level set to 10^6^ for MS level set to 1, and to 0 for MS level set to 2. The ADAP chromatogram builder was used and set to a minimum group size of scans of 5, minimum group intensity threshold of 10^5^, and minimum highest intensity of 10^5^ and *m/z* tolerance of 8.0 ppm. For chromatogram deconvolution, the algorithm used was the wavelets (ADAP). The intensity window S/N was used as S/N estimator with a signal to noise ratio set at 25, a minimum feature height at 10,000, a coefficient area threshold at 100, a peak duration ranges from 0.02 to 0.9 min and the RT wavelet range from 0.02 to 0.05 min. Isotopes were detected using the isotopes peaks grouper with a *m/z* tolerance of 5.0 ppm, a RT tolerance of 0.02 min (absolute), the maximum charge set at 2 and the representative isotope used was the most intense. An adduct (Na^+^, K^+^, NH_4_^+^, CH_3_CN^+^, CH_3_OH^+^, C_3_H_8_O^+^, IPA^+^) search was performed with the RT tolerance set at 0.1 min and the maximum relative peak height at 500%. A complex search was also performed using [M + H]^+^ for ESI positive mode, with the RT tolerance set at 0.1 min and the maximum relative peak height at 500%. Peak alignment was performed using the join aligner method (*m/z* tolerance at 8 ppm), absolute RT tolerance 0.065 min, weight for *m/z* at 10 and weight for RT at 10. The peak list was gap-filled with the same RT and *m/z* range gap filler (*m/z* tolerance at 8 ppm). Eventually, the resulting aligned peak list was filtered using the peak-list rows filter option in order to keep only features associated with MS2 scans^53^.

For the construction of the network, PI strains were not kept and only PP, PF, PCM and PC strains are displayed.

In order to keep the retention time, the exact mass information and to allow for the separation of isomers, a feature based molecular network (https://ccms-ucsd.github.io/GNPSDocumentation/featurebasedmolecularnetworking/) was created using the .mgf file resulting from the MzMine pretreatment step detailed above. Spectral data was uploaded on the GNPS molecular networking platform. A network was then created where edges were filtered to have a cosine score above 0.7 and more than 6 matched peaks. Further edges between two nodes were kept in the network if and only if each of the nodes appeared in each other's respective top 10 most similar nodes. The spectra in the network were then searched against GNPS' spectral libraries. All matches kept between network spectra and library spectra were required to have a score above 0.7 and at least 6 matched peaks. The output was visualised using Cytoscape 3.6 software^56^. The GNPS job parameters and resulting data are available at the following address (https://gnps.ucsd.edu/ProteoSAFe/status.jsp?task=e81d79e32ab848d5917206961e0cf8ad).

### Ethics declarations

All strains and clinical information used in this study were collected as part of the periodic monitoring of patients at the Hospices Civils de Lyon. Bacterial strains are not considered as human biological samples by French regulations, so their reuse for research purposes does not require prior information to or consent from the patient. As the study is retrospective and non-interventional neither ethics committee approval nor written informed consent were required within local regulations. In accordance to European and French data protection regulations, we use an irreversible anonymization making any identification of patient impossible.

## Supplementary Information

Supplementary Information.
