# Supplementary material for: Alkyl-Quinolones derivatives as potential biomarkers for Pseudomonas aeruginosa infection chronicity in Cystic Fibrosis
Source: Sci Rep. 2021 Oct 20;11:20722. doi: 10.1038/s41598-021-99467-7 (PMC8528811; doi:10.1038/s41598-021-99467-7)
Supplement: Supplementary file 1 — Supplementary Information. [file 41598_2021_99467_MOESM1_ESM.docx]

**lkyl-Quinolones derivatives as potential biomarkers for *Pseudomonas* *aeruginosa* infection chronicity in Cystic Fibrosis**

Serge Michalet^1,2,3^, Pierre-Marie Allard^4^, Carine Commun^1,2,5^, Van Thanh Nguyen Ngoc^1,2,3^, Kodjo Nouwade^1,2,3^, Bruna Gioia^6^, Marie-Geneviève Dijoux-Franca^1,2,3^, Jean-Luc Wolfender^4^ and Anne Doléans-Jordheim A^1,2,5,7*^

**Figure S1 :** PFGE profiles (Dice UPGMA tolerance 1.5%) for all *Pa* strains used in our study.

**Figure S2 :** Full metabolic network corresponding to the analysis of chronic (PC/PCM, n = 20) and first colonisation (PP/PF, n = 19) strains.


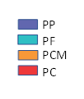


**Figure S3 :** HQ/dbHQ cluster : PP (strains from patients never colonised by *Pa*; dark blue), PF (strains from patients not colonised by *Pa* during at least one year before; blue); PC and PCM (patient chronically colonised by *Pa*; red and orange respectively)


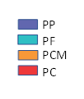


**Figure S4 :** dbHQ(Δ1) cluster : PP (strains from patients never colonised by *Pa*; dark blue), PF (strains from patients not colonised by *Pa* during at least one year before; blue); PC and PCM (patient chronically colonised by *Pa*; red and orange respectively)


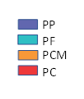


**Figure S5:** PQS/dbPQS cluster: PP (strains from patients never colonised by *Pa*; dark blue), PF (strains from patients not colonised by *Pa* during at least one year before; blue); PC and PCM (patient chronically colonised by *Pa*; red and orange respectively)


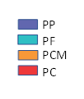


**Fig. S6:** HQNO/dbHQNO(Δ1) cluster: PP (strains from patients never colonised by *Pa*; dark blue), PF (strains from patients not colonised by *Pa* during at least one year before; blue), PC and PCM (patient chronically colonised by *Pa*; red and orange respectively)
